# The effect of CETP inhibitors on new-onset diabetes: a systematic review and meta-analysis

**DOI:** 10.1093/ehjcvp/pvac025

**Published:** 2022-04-20

**Authors:** Katerina Dangas, Ann-Marie Navar, John J P Kastelein

**Affiliations:** Magdalen College University of Oxford, Oxford, OX1 4AU UK; Department of Internal Medicine, UT Southwestern Medical Center, Dallas, TX 75390, USA; Department of Vascular Medicine, Amsterdam UMC, University of Amsterdam, Amsterdam 1081, Netherlands

## Abstract

**Background:**

Despite the increasing prevalence of type 2 diabetes mellitus (T2DM), limited pharmacologic options are available for prevention. Cholesteryl ester transfer protein inhibitors (CETPis) have been studied primarily as a therapy to reduce cardiovascular disease, but have also been shown to reduce new-onset diabetes. As new trial data have become available, this meta-analysis examines the effect of CETP inhibitors on new-onset diabetes and related glycaemic measures.

**Methods and results:**

We searched MEDLINE, EMBASE, and Cochrane databases (all articles until 4 March, 2021) for randomised controlled trials (RCT) ≥1-year duration, with at least 500 participants, comparing CETPi to placebo, and that reported data on new-onset diabetes or related glycaemic measures [haemoglobin A1C (HbA1C), fasting plasma glucose, insulin, Homeostatic Model Assessment of Insulin Resistance (HOMA-IR)]. A fixed effects meta-analysis model was applied to all eligible studies to quantify the effect of CETPi therapy on new-onset diabetes. Four RCTs (*n* = 75 102) were eligible for quantitative analysis of the effect of CETPi on new-onset diabetes. CETPis were found to significantly decrease the risk of new-onset diabetes by 16% (RR: 0.84; 95% CI: 0.78, 0.91; *P* < 0.001), with low between-trial heterogeneity (I_2_ = 4.1%). Glycaemic measures were also significantly improved or trended towards improvement in those with and without diabetes across most trials.

**Conclusion:**

Although RCTs have shown mixed results regarding the impact of CETPi on cardiovascular disease, they have shown a consistent reduction in the risk of new-onset diabetes with CETPi therapy. Future trials of CETPis and potentially other HDL-raising agents should therefore specify new-onset diabetes and reversal of existing T2DM as secondary endpoints.

## Introduction

Type 2 Diabetes Mellitus (T2DM)^1^ is growing exponentially. T2DM nearly doubles the risk of coronary heart disease (CHD) and stroke, and correlates with worse prognosis and increased cardiovascular disease (CVD) mortality.^1^ The impact of T2DM on CVD is partly mediated through diabetic dyslipidaemia, characterised by low high-density lipoprotein (HDL), hypertriglyceridaemia, and increased Apo-B levels.

Unfortunately, beyond lifestyle, pharmacologic therapies to prevent the onset of diabetes are limited. Metformin can lower the risk of diabetes in those with prediabetes.^2^ Emerging T2DM therapies, sodium-glucose co-transporter-2 (SGLT2) inhibitors and glucagon-like peptide-1 (GLP-1) agonists, may improve glycaemic control,^3,4^ but have not yet been evaluated for T2DM prevention. Statins are a cornerstone of cardiovascular risk reduction in persons with and without diabetes, but have been shown to increase the risk of new-onset T2DM.^5,6^

**Figure 1:**
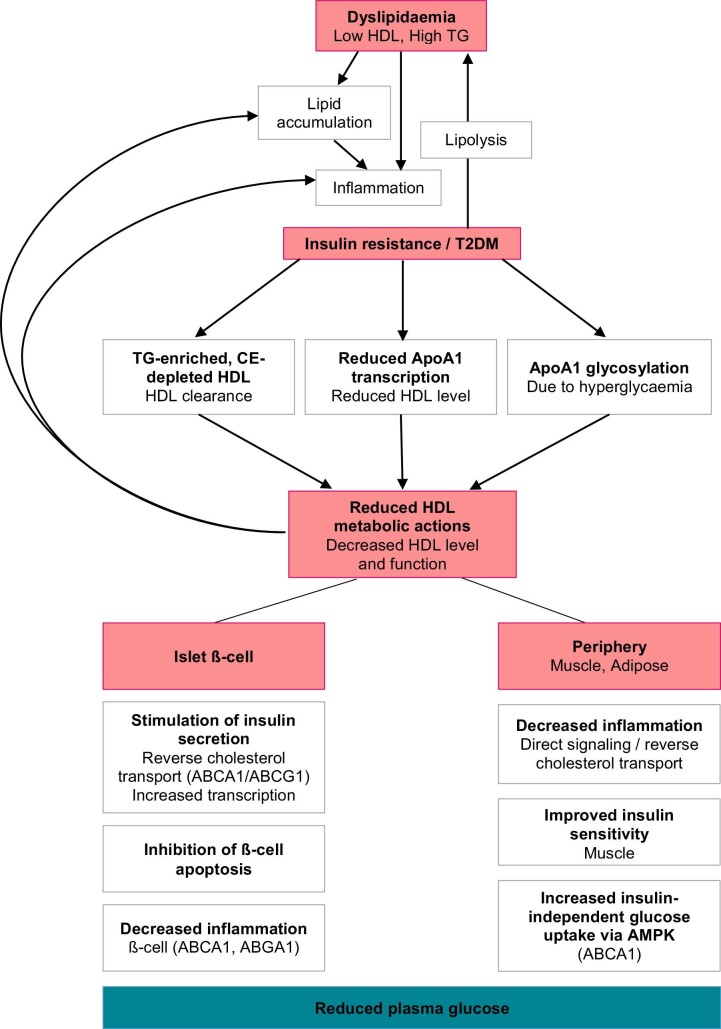
Mechanisms linking HDL-C and diabetes. Dyslipidaemia (low HDL) causes lipid accumulation and inflammation propagating insulin resistance and T2DM. T2DM increases ApoA1 glycation, reduces ApoA1 transcription and changes HDL composition to increase clearance, reducing HDL level and function. HDL normally has reverse cholesterol transport and anti-inflammatory actions at ß-cells and in periphery that decrease plasma glucose.^7^ HDL promotes insulin secretion via ApoA1^30^ and ABCA1/ABCG (1) or through stimulating insulin transcription. HDL-C may inhibit ER-stress-induced ß-cell apoptosis^31^ and islet cell inflammation via ABCA1 and ABCG.^32^ Loss of HDL particles and function exacerbates lipid accumulation and inflammation and increases plasma glucose, contributing to a vicious cycle.

Cholesteryl ester transfer protein inhibitors (CETPis) are a class of lipid lowering medications with potential to lower the risk of new onset diabetes. Cholesteryl ester transfer protein (CETP) promotes exchange of triglycerides (TGs) and cholesterol ester (CE) from HDL to atherogenic ApoB100-containing lipoproteins. CETP inhibition also increases cholesterol-efflux from peripheral tissues, raising HDL-cholesterol (HDL-C) by shifting the partitioning of cholesterol towards HDL particles^7^ (*Figure 1*, Appendix 8). Unfortunately, randomized controlled trial (RCT) data have largely failed to show a benefit for CETPis as a class, with only one large RCT showing cardiovascular benefit from CETP inhibition,^8^ and several other studies showing no effect.^9–12^ A recent Mendelian randomization analysis points to compound specific failures, but not to a lack of class effect on cardiovascular outcomes.^13^ In fact, genetic work suggests that the impact on major cardiac events may depend mainly on absolute LDL-C and time of trial, as with other LDL-C lowering therapies.^14^

Interestingly, some CETPi trials have trended towards reduction in new-onset diabetes. Biologic plausibility exists for the ability to prevent diabetes through CETP inhibition: Individuals with CETP polymorphisms that increase HDL-C have been shown to have lowered risk of T2DM and improved glycaemic status.^15^ Cellular and mechanistic studies also support a potential mechanistic link between CETP, HDL-C, and T2DM.^16,17^

Most initial RCTs of CETPi did not pre-specify or report on new-onset diabetes as an outcome, with the exception of randomized evaluation of the effects of anacetrapib through lipid modification (REVEAL) (anacetrapib vs. placebo, *n* = 30 449), which did find a reduction in new-onset diabetes with anacetrapib therapy.^8^ One prior meta-analysis of 4 RCTs evaluating the link between CETPi and new-onset diabetes^18^ found that CETPis reduced incidence of new-onset diabetes by 12%. Since its publication, novel post-hoc analyses of major trials, assessment of clinical effects of cholesteryl ester transfer protein inhibition with evacetrapib in patients with a high risk for vascular outcomes (ACCELERATE) (evacetrapib vs. placebo) and Dal-Outcomes (dalcetrapib vs. placebo) have been published^19,20^ with additional data now released on new-onset diabetes and glycaemic measures.

Hence, this meta-analysis sought to use all available large randomized clinical trial data available to evaluate the effect of CETPi on new-onset diabetes. Further, we sought to determine whether changes in related measures [haemoglobin A1C (HbA1C), fasting plasma glucose, insulin, Homeostatic Model Assessment of Insulin Resistance (HOMA-IR)] were concordant with any observed effect.

## Methods

MEDLINE, Embase, and Cochrane databases were systematically searched (all articles prior to 4 March, 2021) to identify results from randomised controlled trials (RCTs) that assess efficacy or safety of CETPi against placebo. Search strategies (Appendix 2) were constructed with drug names (obicetrapib, evacetrapib, dalcetrapib, anacetrapib, torcetrapib). McMaster RCT-hedge was adapted to restrict MEDLINE and Embase searches to RCTs.^21^ Reference lists of identified trials and ClinicalTrials.gov were also searched for additional relevant RCTs.

Title and abstract were screened independently. Full text was extracted from chosen publications and subsequently evaluated. Inclusion criteria were (a) RCTs comparing CETPi to placebo (b) participants were adults (c) not reviews or editorials (d) planned treatment period >1-year (e) >500 participants and (f) reported on one of new-onset diabetes, HbA1C, plasma glucose, insulin, or HOMA-IR. Conference abstracts were excluded if published manuscript was included. Quantitative analysis was limited to studies reporting new-onset diabetes.

Study characteristics, patient characteristics (age, sex, diabetes, statin use), and HDL-C and low-density lipoprotein cholesterol (LDL-C) at baseline and at >1-year were extracted (*Tables 1*, *3*). The definition of new-onset diabetes and all data on new-onset diabetes, HbA1C, fasting plasma glucose, and insulin were also extracted. Quality of included studies was assessed independently by two reviewers using Cochrane Collaboration's tool for assessing risk of bias.^22^ Manuscript screening and quality assessment were done by K.D. and a senior consultant at the University of Oxford. Data extraction and analysis was done by K.D.

**Table 1:** Characteristics of randomized controlled trials comparing effect of CETP inhibitor (CETPi) to placebo

			Trial	Enrollment		Size	Arm (n)				Inclusion	Diabetes at baseline (%)		New-onset diabetes (n)		New-onset diabetes criteria
Study	Year	Drug	Type	sites	Duration	(n)	Treatment				Criteria					
							CETPi	Placebo	CETPi	Placebo		CETPi	Placebo	CETPi	Placebo	
ILLUMINATE^9^	2007	Torcetrapib	R, DB, PC, P	221 sites in NA, Eu, Aus	4.5y^†^	15067	7533	7534	60 mg CETPi with Atorvastatin	Placebo with Atorvastatin	45–75 yo with history CVD 1–5 mo prior or T2DM who met ADA criteria or on hypoglycemic therapy	43.5	45.2	76	96	Not reported.
Dal-OUTCOMES^12^	2012	Dalcetrapib	R, DB, PC, P	27 countries in NA, Eu, Asia, Aus	31 mos*	15871	7938	7933	600 mg CETPi with Standard of Care^‡^	Placebo with Standard of Care	>45 yo with recent ACS, has completed planned coronary revascularization procedures	24	25	403	516	Post-randomization diabetes-related adverse event, new use of antihyperglycaemic medication, haemoglobin A1c ≥6.5%, or a combination of at least two measurements of serum glucose ≥7.0 mmol/L (fasting) or ≥11.1 mmol/L (random) for a person without evidence of diabetes mellitus at baseline.
ACCELERATE^10^	2017	Evacetrapib	R, DB, PC, P	36 countries in NA, SA, Eu, Asia, Aus	26 mos*	12092	6038	6054	130 mg CETPi with Standard of Care^‡^	Placebo with Standard of Care	>18 yo with high risk vascular disease, treated with statin 1 month pre-screening	68.4	67.9	175	200	Fasting plasma glucose ≥7 mmol/L or 2-hour plasma glucose ≥11.1 mmol/L during an oral glucose tolerance test or HbA1c levels ≥6.5% (≥48 mmol/mol). Confirm by repeat testing on a second day.
REVEAL^8^	2017	Anacetrapib	R, DB, PC, P	41 sites in NA, Eu, China	4.1 y	30449	15225	15224	100 mg CETPi with Atorvastatin	Placebo with Atorvastatin	>50 yo with high CV risk	37.1	37.2	510	571	Post-randomization diabetes-related adverse event or the use of antihyperglycaemic medication (insulin or oral treatment) recorded on at least one follow-up visit form for a person without evidence of diabetes mellitus at baseline.
DEFINE^11^	2010	Anacetrapib	R, DB, PC, P	20 countries in NA, Eu, Asia, Aus	18 mos	1623	811	812	100 mg CETPi with Atorvastatin	Placebo with Atorvastatin	18–80 yo with CHD or high risk of CHD	53	53.2	NA	NA	NA

R, randomised; DB, double-blind; PC, placebo-controlled; P, parallel; CO, crossover; NA, North America; Eu, Europe; Aus, Australia; SOC, Standard of Care; ADA, American Diabetes Association; ACS, acute coronary syndrome; CV, cardiovascular; CHD, coronary heart disease; CVD, cardiovascular disease; T2DM, Type 2 Diabetes Mellitus.

*Median.

^†^Study was terminated at 1 year.

^‡^Standard of Care is statin therapy.

To evaluate potential association of CETPi and new-onset diabetes, risk ratios (RR) and 95% confidence intervals (CI) were calculated from available data for patients without T2DM at baseline and those who developed T2DM in follow-up. RRs were pooled using a fixed-effects meta-analysis model. All analyses were conducted using STATA (College Station, TX, USA). All analyses were planned and conducted in accordance with preferred reporting items for systematic reviews and meta-analyses checklist (Appendix 7).

## Results

### Search results and characteristics of included trials

Electronic search identified 1716 relevant publications (Appendix 3), of which 57 underwent full-text review. Five RCTs met inclusion criteria^8–12^ and were included, along with all relevant post-hoc analyses,^19,20,23^ though one of the trials determining the efficacy and tolerability of CETP inhibition with anacetrapib (DEFINE) did not report new-onset diabetes, and was thus excluded from quantitative meta-analysis.^8–10^,^12^ All included trials had low risk of bias (Appendix 4).

*Table 1* shows characteristics of CETPi trials in this meta-analysis, which included 75 102 persons with CVD or at high risk of CVD randomized to CETPi vs. placebo. The average duration of follow-up ranged from 1.5 to 4.5y (*Table 1*). CETP inhibitors evaluated were torcetrapib, dalcetrapib, evacetrapib, and anacetrapib; no studies of obicetrapib (TA-8995) met our inclusion criteria. *Table 2* shows patient characteristics in those included in the trials. The mean age of participants was between 60–67 years, over 75% were male, and nearly all (96.4% or higher) were on a statin at baseline.

**Table 2:** Baseline characteristics of patients enrolled in each arm of trials comparing effect of CETPi to placebo

		Arm (n)		Mean Age (SD)		Male sex (%)		Diabetes (%)		Mean BMI (SD)		Statin use (%)	
Study	Drug	CETPi	Placebo	CETPi	Placebo	CETPi	Placebo	CETPi	Placebo	CETPi	Placebo	CETPi	Placebo
ILLUMINATE^9^	Torcetrapib	7533	7534	61.3±7.6	61.3±7.6	77.7	77.8	43.5	45.2	30.1±5.7	30.2±5.6	100	100
Dal-OUTCOMES^12^	Dalcetrapib	7938	7933	60.3±9.1	60.1±9.1	80	81	24	25	28.6±5.0	28.6±5.1	97	98
ACCELERATE^10^	Evacetrapib	6038	6054	64.8±9.4	65.0+9.5	77	77	68.4	67.9	NR	NR	96.4	96.6
REVEAL^8^	Anacetrapib	15225	15224	67±8	67±8	83.9	83.8	37.1	37.2	28.6±5.0	28.6±5.1	97.2	96.9
DEFINE^11^	Anacetrapib	811	812	62.5±8.7	62.9±9.0	77.6	76.1	53	53.2	30.4±5.5	30.1±5.2	99.5	99.1

BMI, body mass index; CETPi, CETP inhibitor.

Lipid measures at baseline and on-treatment are summarized in *Table 3*. The baseline LDL-C ranged from 61 to 82 mg/dL, and baseline HDL-C ranged from 40–49 mg/dL. The effects of CETPi on HDL-C varied by agent, with the greatest increases in HDL-C seen with anacetrapib and evacetrapib. All reduced LDL-C by 20–30% relative to placebo except dalcetrapib which had minimal effect on LDL-C (*Table 3*).

**Table 3:** Effect of CETPi on lipids

				LDL-C (mg/dL)							HDL-C (mg/dL)						
Study	Drug	Arm (n)		Baseline (SD)		>1 year (SD)		Change from baseline (%)			Baseline (SD)		>1 year (SD)		Change from baseline (%)		
		CETPi	Placebo	CETPi	Placebo	CETPi	Placebo	CETPi	Placebo	CETPi-Placebo (%)	CETPi	Placebo	CETPi	Placebo	CETPi	Placebo	CETPi-Placebo (%)
ILLUMINATE^9^	Torcetrapib	7533	7534	79.7 (20.4)	79.9 (20.4)	NR	NR	–24.9	3.0	–27.9%^†^	48.6 (12.0)	48.5 (12.2)	NR	NR	72.1	1.8	70.3%^†^
Dal-OUTCOMES^12^	Dalcetrapib	7938	7933	76.4 (26.4)	75.8 (25.9)	NR	NR	NR	NR	0.0%	42.5 (11.7)	42.2 (11.5)	58.6 (NR)	46.1 (NR)	40.0	11.0	29%^†^
ACCELERATE^10^	Evacetrapib	6038	6054	81.6 (28.4)	81.1 (27.8)	54.7 (26.4)	83.7 (30.8)	–31.1	6.0	–37.1%	45.3 (11.7)	45.3 (11.7)	104.1 (31.4)	45.6 (12.3)	133.2	1.6	131.6%
REVEAL^8^	Anacetrapib	15225	15224	61 (15)	61 (15)	38 (NR)	64 (NR)	NR	NR	–41.0%	40 (NR)	40 (NR)	85 (NR)	42 (NR)	NR	NR	104.0%
DEFINE^11^	Anacetrapib	811	812	81.2 (21.3)	82.2 (20.7)	48.9 (NR)	76.7 (NR)	–40.5	–4.3	–36.2%	40.5 (9.3)	40.4 (9.1)	102.3 (NR)	44.9 (NR)	151.1	12.3	138.8%

*ACCELERATE only reported lipid levels at 3 months.

^†^Calculated based on available data.

### Impact of cholesteryl ester transfer protein inhibitors on new onset diabetes

New-onset diabetes was defined as the development of diabetes after initiation of the pharmacological intervention in patients who were not diabetic at baseline. The precise criteria varied between trials (*Table 1*). While REVEAL based diagnosis on physician reporting,^8^ ACCELERATE diagnosed with biomarkers,^10^ and dal-OUTCOMES with both.^12^ The rates of diabetes in the included populations at baseline varied from 24% in Dal-OUTCOMES to 68.4% in ACCELERATE. A total of 41 739 persons free of diabetes were included in the four trials available for quantitative meta-analysis.

**Figure 2:**
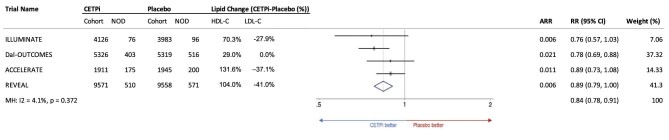
Forest plot indicating reduction in risk of new-onset diabetes with CETP inhibitor therapy in using a fixed-effects model (weights are from Mantel–Haenszel model). Included ILLUMINATE,^23^ dal-OUTCOMES,^20^ ACCELERATE^19^ and REVEAL.^8^ RR, risk ratio; NOD, new-onset diabetes.

*Figure 2* shows results from individual trials and pooled results from the meta-analysis on the risk of new onset diabetes among those free of diabetes at baseline. All four trials reported lower rates of new onset diabetes among those given CETPi vs. placebo, though this was only statistically significant in Dal-OUTCOMES. In pooled analysis, CETPi therapy was associated with a reduced risk of new onset diabetes relative to placebo (RR: 0.84; 95% CI: 0.78–0.91; *P* <0.001) (*Figure 2*), with low between-study heterogeneity (I_2_ = 4.1%). Absolute risk reduction ranged from 0.6–2.1% with CETPi therapy (*Figure 2*). No indication of publication bias was observed for new-onset diabetes according to the funnel plot (Appendix 5).

### Impact of cholesteryl ester transfer protein inhibitors on glycaemic measures in those with diabetes

All but one trial (dal-OUTCOMES) reported showed differences in haemoglobin A1c between placebo and treatment arms among those with diabetes (*Table 4*). Investigation of lipid level management to understand its impact in atherosclerotic events (ILLUMINATE) and ACCELERATE each demonstrated a statistically significant reduction in HbA1c for Torcetrapib and Evacetrapib, respectively, while no difference was seen in HbA1c in REVEAL or DEFINE for anacetrapib. In the ILLUMINATE trial, within the cohort of persons with diabetes, patients in placebo group were more frequently given add-on insulin [200 patients (5.9%) in placebo and 165 patients (5.04%) in torcetrapib; *P* = 0.13] and oral antidiabetic therapy [456 patients (13.45%) in placebo and 394 (12.05%); *P* = 0.09] than in torcetrapib group, potentially suggesting that the true effect of torcetrapib on glucose homeostasis may have been even stronger.^23^

**Table 4:** Glycemic measures

		Persons with diabetes				Persons without diabetes			
Study	Drug	HbA1C	Plasma Glucose	Insulin	HOMA-IR	HbA1C	Plasma glucose	Insulin	HOMA-IR
ILLUMINATE^23^	Torcetrapib	At 12 mo: placebo arm (n = 2999) 7.36%; CETPi arm (n = 2796) 7.16% (*P* < 0.001)	At 3 mo: 0.34 mmol/L lower in CETPi arm (n = 3137) than placebo arm (n = 3288) (*P* < 0.001)	At 3 mo: 11.7 μU/mL lower in CETPi arm (n = 3092) than placebo arm (n = 3230) (*P* < 0.0001)	At 3 mo: Decreased from 49.1 to 47.3 in CETPi arm (n = 3053) (*P* < 0.0001), increase in placebo arm (n = 3200)	At 12 mo: placebo arm (n = 240) 6.52%; CETPi (n = 219) 6.27% (p<0.001)	At 12 mo: –0.06 Placebo (n = 3857)-CETPi (n = 3972) (*P* = 0.03)	At 3 mo: 6.6 lower in CETPi arm (n = 3092) than placebo arm (n = 3947) (*P* < 0.001)	At 3 mo: Decreased from 22.35 to 21.89 in CETPi arm (n = 4026) while increase in placebo arm (n = 3906)
Dal-OUTCOMES^20^	Dalcetrapib	Not reported	Not reported	Not reported	Not reported	At 12 mo: placebo arm (n = 6923) 5.9%; CETPi (n = 6847) 5.8%, (*P* < 0.01)*	At 12 mo: 5.6 mmol/L in placebo arm (n = 5397); CETPi arm (n = 5297) (*P* > 0.05)*	At 3 mo: –0.61 μU/mL CETPi (n = 1071)-Placebo (n = 1097) (*P* > 0.05)	At 3 mo: Placebo (n = 1097) 2.09; CETPi (n = 1071) 1.95 (*P* > 0.05)
ACCELERATE^19^	Evacetrapib	At 6 mo: placebo arm 7.15%; CETPi arm 7.08% (*P* = 0.023)	Not reported	Not reported	Not reported	Not reported	Not reported	Not reported	Not reported
REVEAL^8^	Anacetrapib	Placebo arm 6.9%; CETPi arm 6.9% (*P* = 0.92) (n = NR)^†^	Not reported	Not reported	Not reported	–0.03 CETPi-placebo (*P* < 0.001) (n = NR)^†^	Not reported	Not reported	Not reported
DEFINE^11^	Anacetrapib	At 76 w: CETPi-placebo: –0.11 (*P* = 0.083) (n = NR)	Not reported	Not reported	Not reported	Not reported	At 76 w: –2.5 CETPi-Placebo (*P* = 0.11) (n = NR)	Not reported	Not reported

DEFINE showed changes in HbA1C between CETPi and placebo groups, but the results of the two groups were not individually reported. Green = significant improvement, Yellow = trending towards improvement, Red = no effect. HbA1C, haemoglobin A1C; HOMA-IR, Homeostatic model assessment of insulin resistance; DM, baseline cohort of persons with diabetes; NDM, baseline persons without diabetes; NR, not reported.

*This data reflects the whole cohort (DM + NDM), as majority of dal-OUTCOMES cohort is NDM, it is most representative of this group.

^†^Data collected at final study visit, precise timing not reported.

Only ILLUMINATE reported other diabetes-related biomarkers in persons with diabetes. Plasma glucose (fasting), insulin, and HOMA-IR were all lowered at 3 months in the torcetrapib arm compared with the placebo arm (*Table 4*).

Among those free of diabetes at baseline, three trials reported changes in HbA1c, with similar findings as in those with diabetes; torcetrapib, and dalcetrapib, but not anacetrapib, was associated with a reduction in HbA1C. Diabetes-related biomarkers were reported in participants without diabetes for torcetrapib and dalcetrapib. Torcetrapib showed a reduction in plasma fasting glucose, insulin, and HOMA-IR, while dalcetrapib found no statistically significant differences between the treatment and placebo arms in any of these three markers, possibly explained by the modest HDL-C increase conferred by dalcetrapib.

### Additional data reported from cholesteryl ester transfer protein inhibitors trials

Dal-OUTCOMES^20^ further reported on bidirectional transitions among three glycaemic states: normoglycaemia, prediabetes, and diabetes. Prediabetes to diabetes transition was reduced [dalcetrapib: 364/3394 (10.7); placebo: 473/3301 (14.3); *P* < 0.001]. However, normoglycaemia to diabetes transitions were not significantly reduced [dalcetrapib: 39/1932 (2.0); placebo: 43/2018 (2.1); *P* = 0.80]. Normoglycaemia to prediabetes transition was also decreased by dalcetrapib relative to placebo [dalcetrapib: 711/1846 (38.5%); placebo: 826/1915 (43.1%); *P* = 0.004]. Interestingly, dalcetrapib also significantly increased reversal of diabetes to no diabetes [dalcetrapib: 325/2354 (13.8%); placebo: 271/2393 (11.3), *P* = 0.01].

Finally, torcetrapib was shown to increase systolic blood pressure by a mean of 5.4 mmHg vs. 0.9 mmHg in the atorvastatin-only group (*P* < 0.0001) and it was thought that this increase in blood pressure was associated with adverse outcomes.^9^ Other trials showed very modest changes in systolic blood pressure of <1 mmHg, which is unlikely to be clinically significant.^8,10,12^

## Discussion

This meta-analysis indicates that CETPi, when added to statin therapy, resulted in a statistically significant 16% reduction in new-onset diabetes risk in patients with CVD or at high risk of CVD, with a consistent effect across trials of different CETPis. Glycaemic measures were rarely reported, but trended towards supporting this effect, with decreases in haemoglobin A1c among those with pre-existing diabetes.

While, CETPis are associated with decreased risk of diabetes despite reducing LDL-C, other LDL-C lowering agents are mostly associated with increased or no change in T2DM risk.^6^ Niacin has been associated with a moderately increased risk of incident diabetes,^24^ ezetimibe trials have not reported on new-onset diabetes, bempedoic acid trials indicate at most a very mild (<3%) decrease.^25^ Statins have been shown to increase the risk of incident diabetes, with the highest risk among those who had risk factors for diabetes.^5^

### Genetic studies on cholesteryl ester transfer protein inhibitors and diabetes risk

Genetic studies support a role for CETPi in prevention of diabetes, but with some mixed results. CETP loci are most strongly associated with HDL-C, but also LDL-C and TG, resulting in complex associations with gene-sets related to cholesterol metabolism, lipid transport, and foam-cell differentiation, amongst others.^26^ Polymorphisms that increase CETP and decrease HDL-C have been hypothesized to worsen glycaemic status, while polymorphisms that decrease CETP activity and increase HDL-C may improve glycaemic status.^15^ Indeed, in healthy adults the B2 allele at CETP-related Taq1B locus, which affects CETP promotor activity, is associated with increased-HDL-C and reduced insulin resistance,^27^ while the B1B1-genotype is associated with lower HDL-C^28^ and higher insulin resistance and T2DM risk. These results are consistent with improved diabetes-related measures with CETP. However, the largest candidate-gene study$( {n{\rm{\ }} = {\rm{\ }}5601} )$ found no association between presence of CETP SNP rs3764261 (which increases HDL-C) and T2DM in CVD patients.^29^

The most important evidence suggesting that new-onset diabetes reduction is an on-target effect of CETP inhibition comes from a recent large mendelian randomization study of CETP loci on >190 pharmacologically relevant outcomes with 480 698–21 770 samples and over 74 million events. Low CETP haplotypes were found to be causally related to T2DM, with HDL as the mediator.^13^ Importantly, CETP polymorphisms tend to alter HDL-C levels by ≤5% whereas CETPi change HDL-C by up to 130%, which may explain some of the discordance between candidate-gene studies and clinical trials, though it must also be considered that CETPis are acting acutely whereas genetic polymorphisms act chronically.

### Potential mechanism for cholesteryl ester transfer protein inhibitors and diabetes

If the association between CETPi and reduced risk of new onset diabetes is mediated by changes in lipids, it is unlikely to be due to changes in LDL-C. CETPis induce variable magnitudes of lipid changes but all increased HDL-C and all but dalcetrapib decreased LDL-C. Lack of LDL-C reduction with dalcetrapib is attributed to relatively low CETP inhibition (∼30%), as LDL-C reduction is proportional to the degree of CETP inhibition. Despite this difference, dalcetrapib exerted at least as large a reduction in new-onset diabetes as other CETPi (*Figure 2*) and significantly increased T2DM reversal [${\rm{HR\ }} = {\rm{\ }}1.25,95{\rm{\% \ CI}}( {1.06 - 1.49} ),{\rm{\ p}}P{\rm{\ }} = {\rm{\ }}0.01]){\rm{\ }}($20), indicating an overall positive effect on glycaemic transitions.

All CETPis increase HDL-C which may play a role in their association with diabetes. ACCELERATE^10^ and REVEAL,^8^ studying evacetrapib and anacetrapib respectively, did not exhibit the largest reduction in new-onset diabetes despite largest on-treatment increase in HDL-C beyond placebo (>100% baseline) (*Figure 2*). This implies either a non-linear relationship between HDL-C changes and diabetes risk, or a pathway between CETP inhibition and diabetes that is independent of HDL-C. However, these trial-level evaluations of overall HDL-C changes and diabetes risk are likely insufficient to determine if changes in HDL-C mediate or correlate with diabetes risk reduction, which would be best answered with individual-level data. Future research is needed to elucidate the specific mechanisms of the link between CETP inhibition and incident diabetes, including the impact of CETP inhibition on cholesterol efflux from beta-cells, induction of insulin synthesis by beta cells, and increased glucose uptake by muscle.

There is biological plausibility for a role in HDL in T2DM, including significant mechanistic evidence for a bi-directional relationship between dyslipidaemia and T2DM.^16^ Lipid accumulation and inflammation leads to insulin resistance, while T2DM changes HDL composition and reduces ApoA1^16^ exacerbating dyslipidaemia.^7^ (*Figure 1*)

Cellular and rodent studies have characterised multiple HDL glucose-lowering actions insofar as HDL acts centrally at ß-cells to inhibit apoptosis, reduce inflammation and promote insulin secretion, reviewed elsewhere.^7,16^ In the periphery, HDL-mediated cholesterol-efflux increases insulin sensitivity and glucose uptake, especially by muscle.^7^ Together, these mechanisms reduce plasma glucose, suggesting a role for HDL-C in diabetes pathogenesis. Accordingly, in T2DM patients, recombinant-HDL stimulated insulin secretion and reduced plasma glucose.^17^ Interestingly, plasma glucose reduced at 30 min whereas insulin secretion rose after 1.5h, suggesting an insulin-independent glucose-lowering mechanism, potentially AMPK-dependent glucose uptake, which may be enhanced by ApoA1.^16^ This study acutely altered HDL-C, so suggested mechanisms may reflect the impact of chronic changes in HDL-C.

Overall, HDL appears to play a role in glucose metabolism, but the exact chronology and clinical relevance of these mechanisms, and the specific role for CETP in this pathway, remain unclear. On a cellular level, dalcetrapib^7^ may stimulate cholesterol efflux at ß-cells encouraging insulin secretion, and torcetrapib has been shown to do so *in vivo* in rodents,^7^ but evidence remains preliminary.

Of note, our study found that the association between CETPi and new-onset diabetes (RR: 0.84; 95% CI: 0.78–0.91; *P* <0.001) appeared stronger than the association between CETPi and glycaemic control among those who already had diabetes (*Table 4*). Thus, the evidence does not support potential role for CETPi to improve glycaemic control in those who have already developed diabetes. Given this small change in glycaemic indices, it is possible that the mechanism of CETPi on T2DM risk is due to direct effects on beta cell survival. Future work should evaluate the degree to which any reduction in risk of T2DM is mediated by HDL-C, as well as other potential measures of HDL including HDL function.

### Limitations

There are several key limitations to the present analysis. First, analysis of new-onset diabetes was post-hoc (except REVEAL^8^), increasing potential for false positive results. However, the consistency across trials increases our pooled result's credibility, and warrants further study through prospective randomised trials with pre-specification of T2DM as an endpoint. Second, methods for new-onset diabetes diagnosis varied between trials. While REVEAL based diagnosis on physician reporting,^8^ ACCELERATE diagnosed on basis of biochemical lab testing^10^ and dal-OUTCOMES accepted both^12^ (*Table 1*). Importantly, however a sensitivity analysis in dal-OUTCOMES suggested both approaches yielded a similar result,^12,20^ suggesting minimal impact on our overall findings.

Furthermore, there was a lack of individual patient-level data as well as a lack of systematic collection of insulin/glucose measurements and diabetes medication or insulin use, which limited the extent of analysis. Specifically, ILLUMINATE did not report criteria for new onset diabetes. However, the hazard ratio for treatment on new onset diabetes for ILLUMINATE was similar to other trials, and exclusion of this trial from the fixed effects model did not change the overall result.

Finally, all of the trials evaluated the effect of CETP inhibitors when added to a background of high-intensity statin therapy. It is not known, therefore, what the consequences might be of CETP inhibitor monotherapy for the new-onset of diabetes. The impact of the last CETP-inhibitor in clinical development, obicetrapib, is as of yet unknown but is prospectively investigated in a large phase III programme currently underway.

## Conclusions

This meta-analysis indicates that CETPis provide a significant 16% reduction in new-onset diabetes risk. Together with increasing mechanistic and genetic evidence, these results support a role for HDL in T2DM pathogenesis and prevention, though further characterization is needed. Prospective randomized trials are needed to evaluate the role of CETPi as a possible adjunct to statin therapy to improve lipid profile in vulnerable patient populations. Future trials of CETPi and potentially other HDL-raising agents should therefore specify new-onset diabetes and reversal of existing T2DM as secondary endpoints.

## Supplementary Material

pvac025_Supplemental_File

## Data availability

The data was extracted using publically available data from previously published randomized clinina trials. No patient identified data were used in these analyses (8-12, 19, 20, 23).

## Registration information

The review was registered at Research Registry with unique identifier: reviewregistry1280.
