# Supplementary material for: The effect of CETP inhibitors on new-onset diabetes: a systematic review and meta-analysis
Source: Eur Heart J Cardiovasc Pharmacother. 2022 Apr 20;8(6):622–32. doi: 10.1093/ehjcvp/pvac025 (PMC9729761; doi:10.1093/ehjcvp/pvac025)
Supplement: pvac025_Supplemental_File [file pvac025_supplemental_file.docx]

Appendices

Appendix 1: Abbreviations

| **Abbreviations** | |
| --- | --- |
| ABCA1 | ATP Binding Casette transporter A1 (HDL binds ABCA1 for cholesterol efflux) |
| ABCG1 | ATP Binding Casette transporter G1 (HDL binds ABCG1 for cholesterol efflux) |
| ACS | Acute Coronary Syndrome |
| CE | Cholesterol Ester |
| CETP | Cholesteryl Ester Transfer Protein |
| CETPi | CETP inhibitors |
| CVD | Cardiovascular disease |
| FC | Free Cholesterol |
| HDL | High Density Lipoprotein |
| HDL-C | High Density Lipoprotein Cholesterol |
| LDL | Low Density Lipoprotein |
| LDL-C | Low Density Lipoprotein Cholesterol |
| RCT | Randomised Controlled Trial |
| T2DM | Type 2 Diabetes Mellitus |
| TG | Triglyceride |

Appendix 2: Search Strategies

*Search 1: EMBASE (via OVID)*


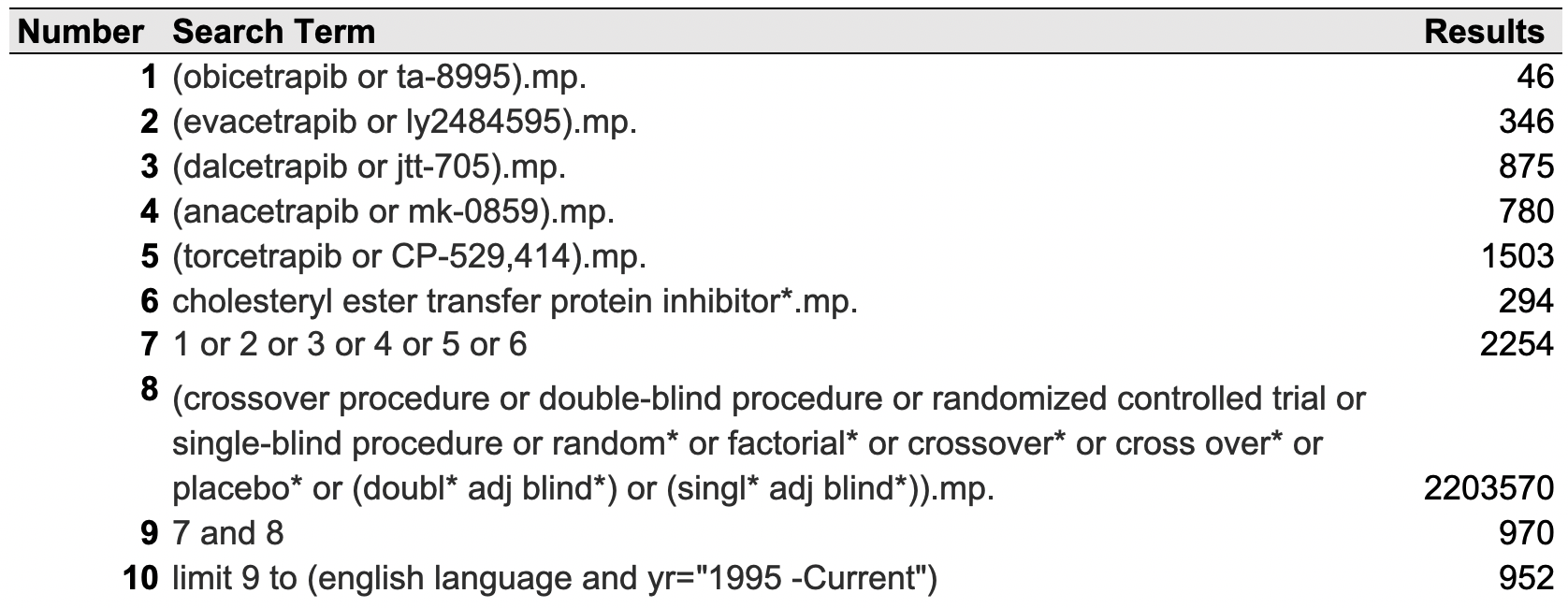


*Search 2: MEDLINE (via OVID)*


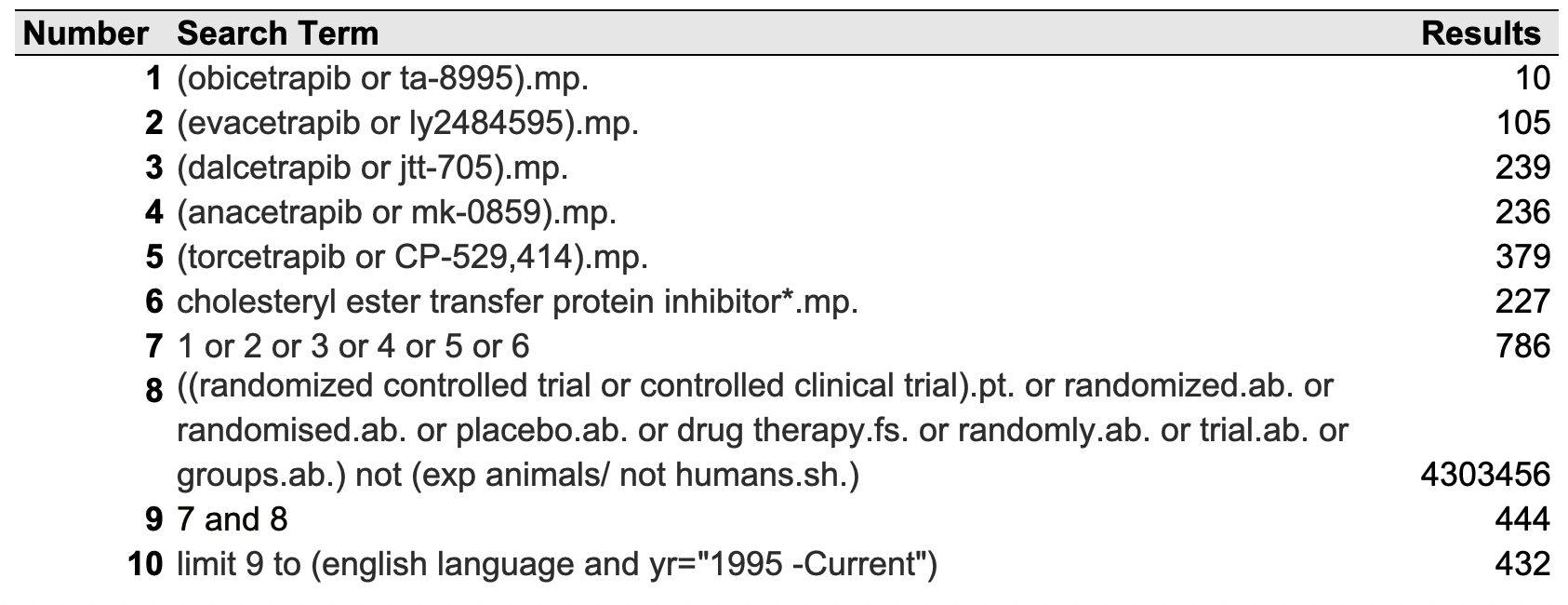


*Search 3: CENTRAL*

**
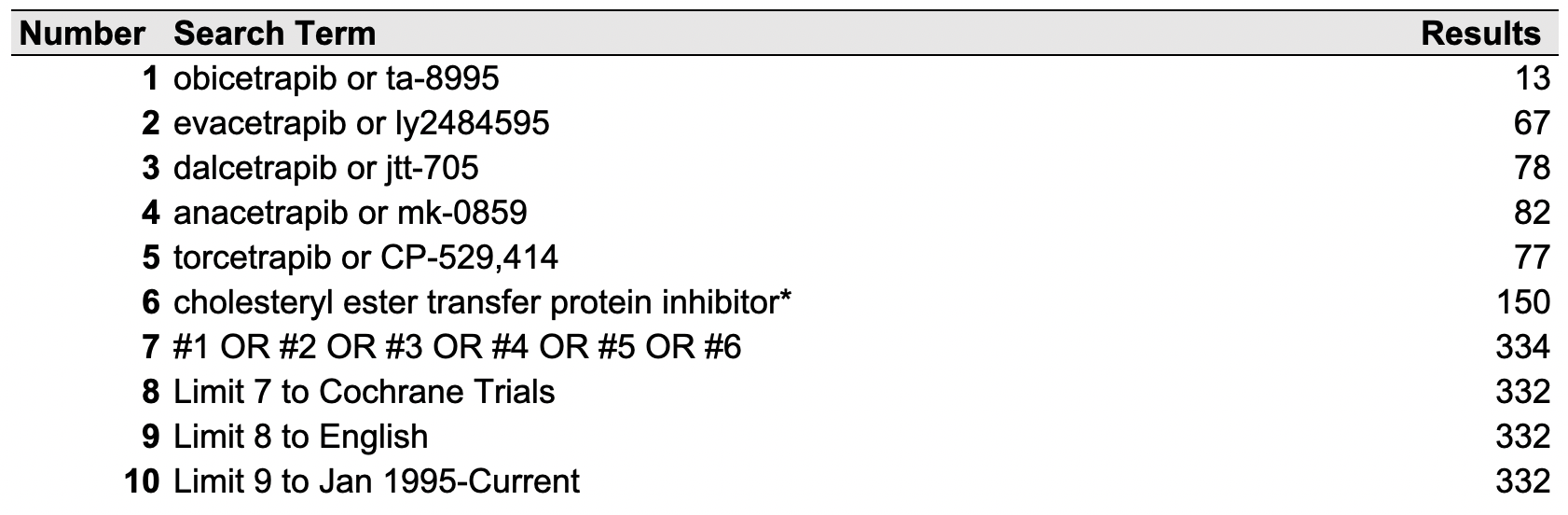
**

Appendix 3: PRISMA Flow Chart

**
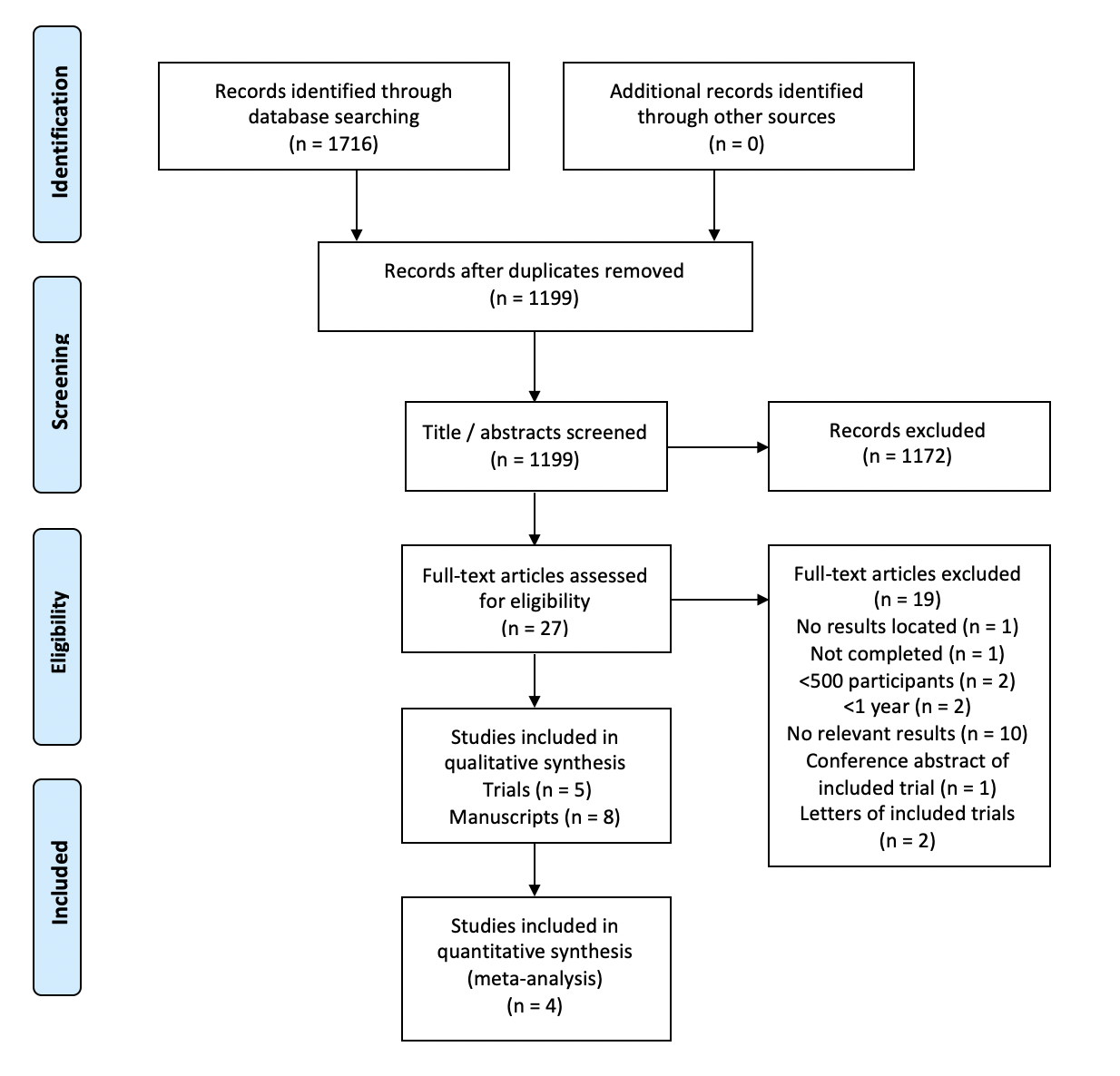
**

This systematic review with meta-analysis followed a pre-specified protocol, designed and reported according to Preferred Reporting Items for Systematic Reviews and Meta-Analyses (PRISMA) guidelines.


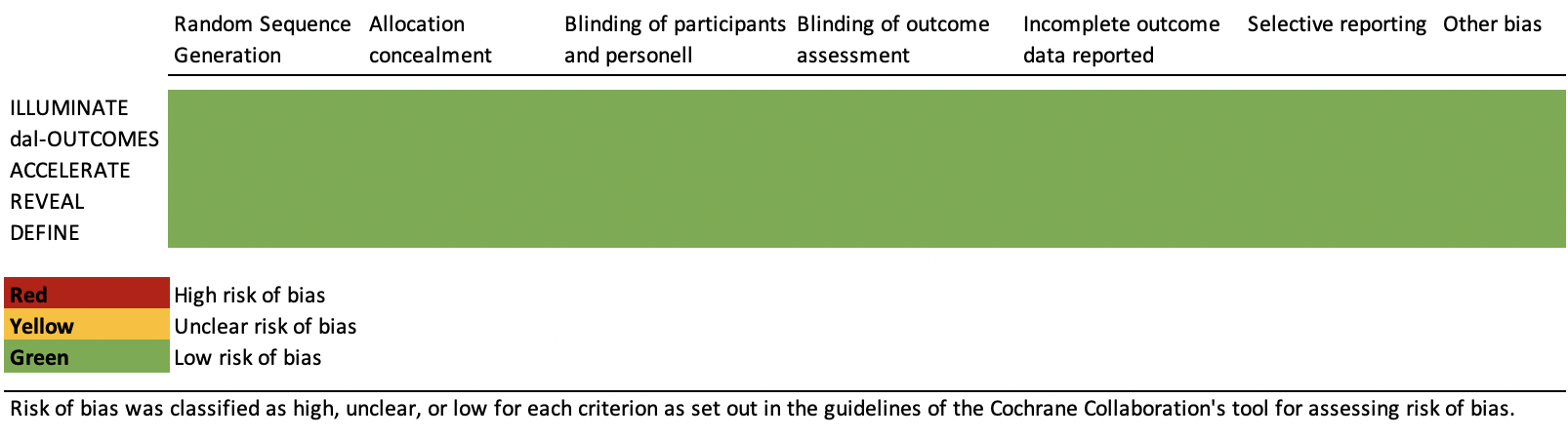
Appendix 4: Quality Assessment Table

Quality of included studies was assessed independently by two reviewers using Cochrane Collaboration’s tool for assessing risk of bias^1^. Disagreements not resolved by consensus were resolved by a third reviewer. Each domain (sequence generation, allocation concealment, blinding of participants, personnel and outcome assessors, incomplete outcome data, selective outcome reporting and other potential sources of bias) was given a score of “high,” “unclear,” or “low”. All five trials included in this meta-analysis were of very high-quality with low risk of bias detected. Trials included were ILLUMINATE^2^, dal-OUTCOMES^3^, ACCELERATE^4^, REVEAL^5^ and DEFINE^6^.

Appendix 5: Funnel plot

**
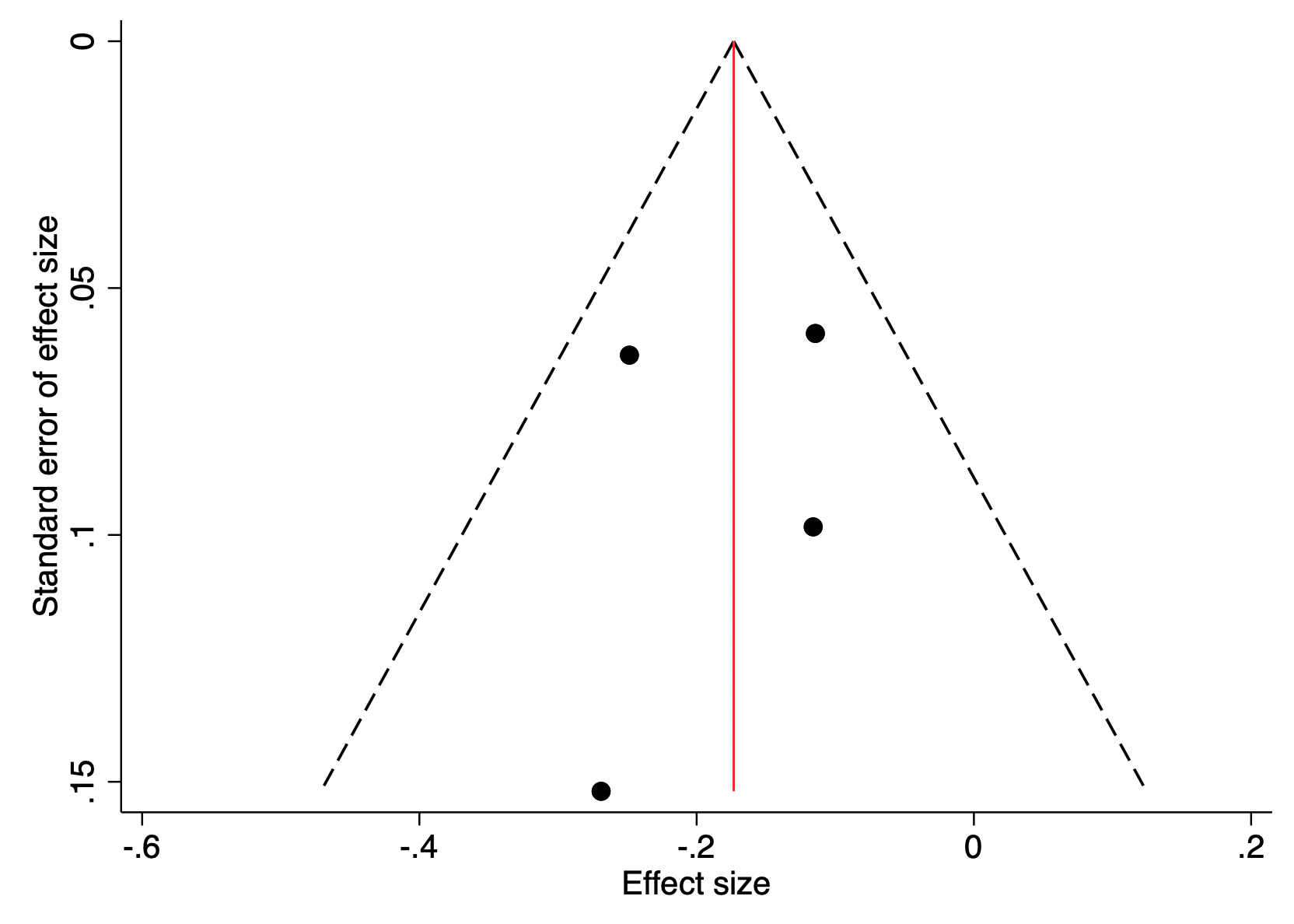
**Funnel plot of effect size for new-onset diabetes indicates no evidence of publication bias.


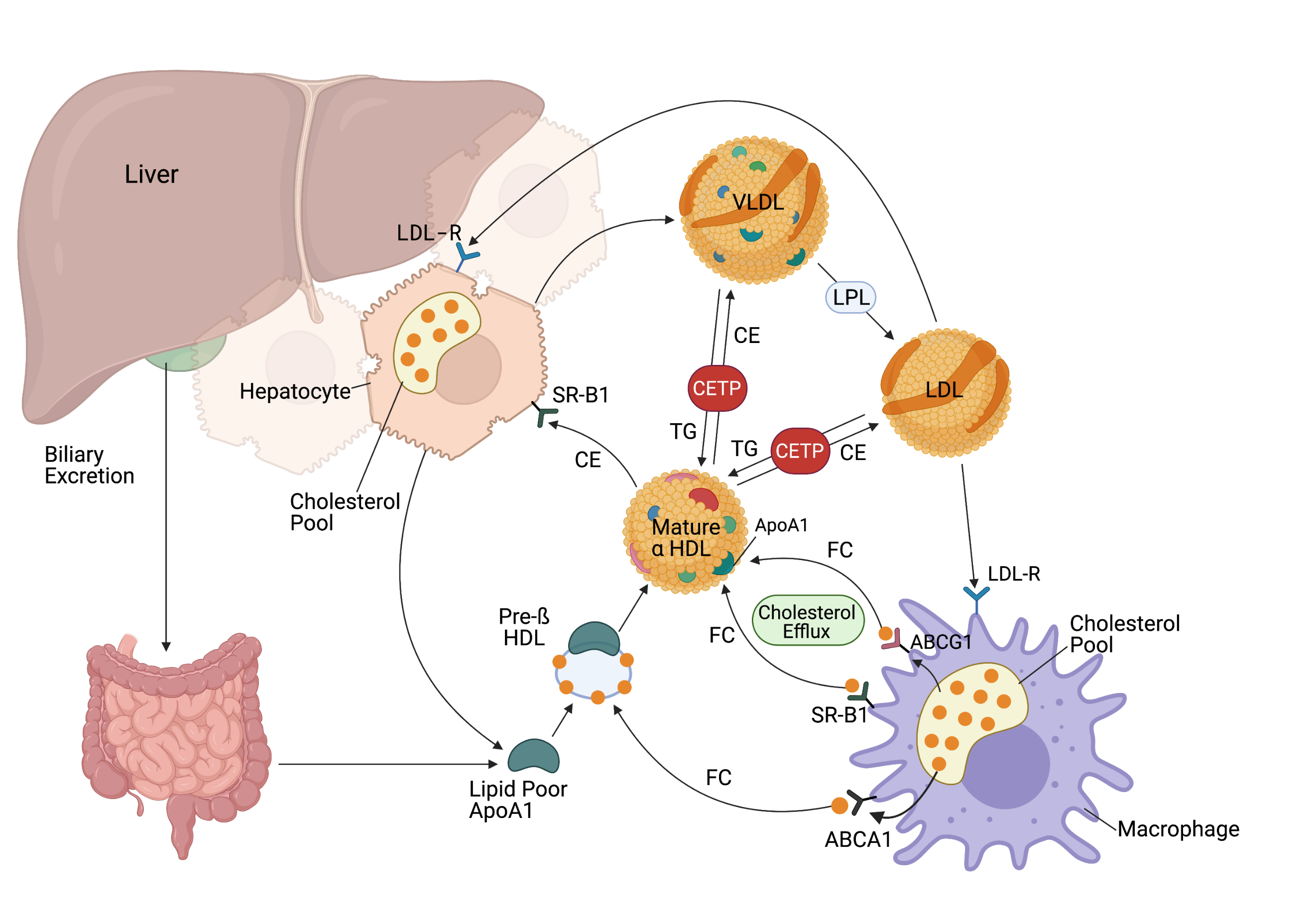
Appendix 6:

CETP mediates transfer of cholesterol ester (CE) from HDL to VLDL/LDL and triglycerides (TG) from VLDL/LDL to HDL. As HDL matures, it amasses free cholesterol (FC) from macrophages in periphery via ABCA1, SR-B1, and ABCG1 and deposits CE at hepatocytes via SR-B1. CETP inhibition thus increases HDL-C, stimulating cholesterol-efflux, and lowers LDL-C decreasing peripheral cholesterol deposits. Created with Biorender.com.

Appendix 7: PRISMA Checklist


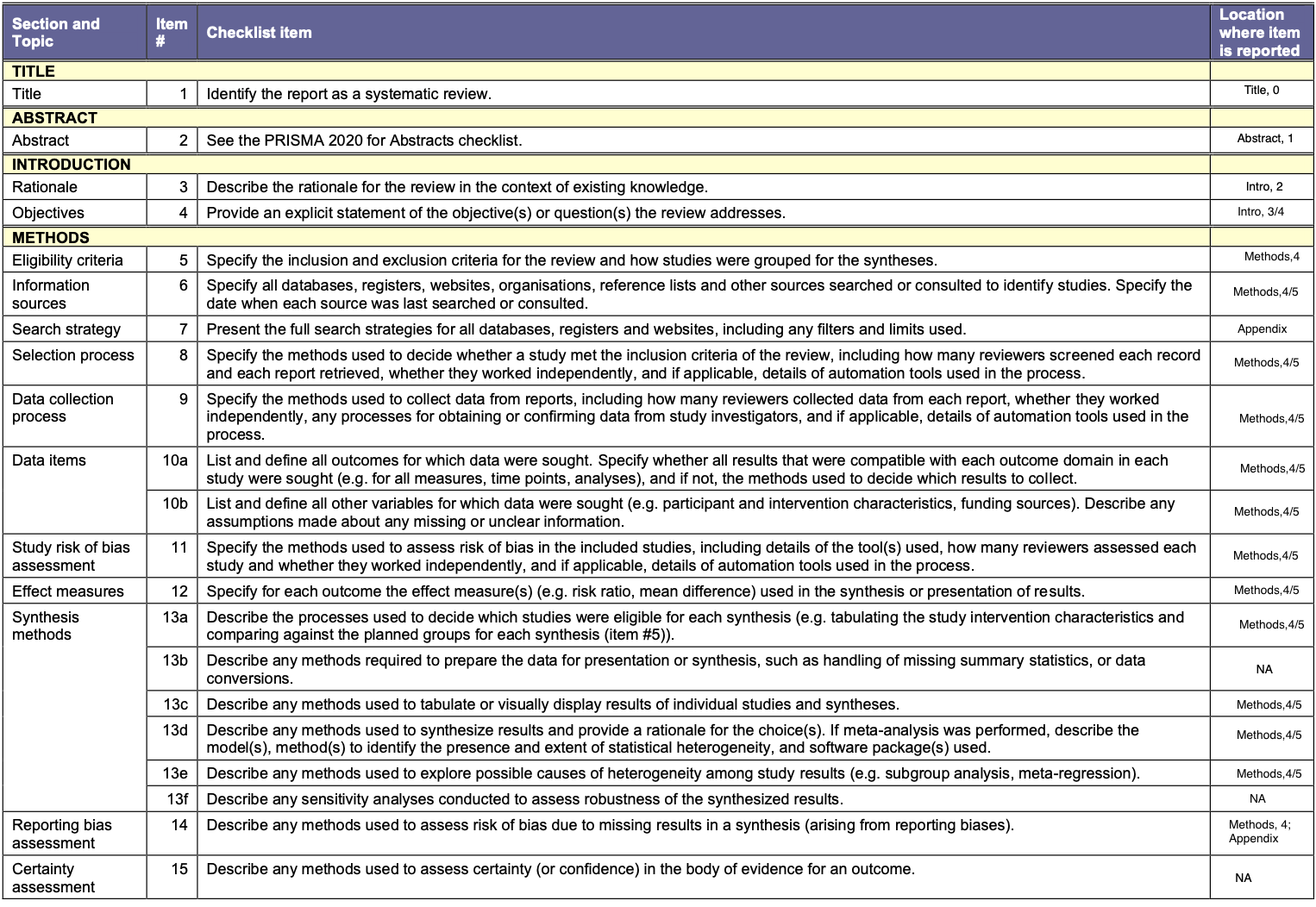


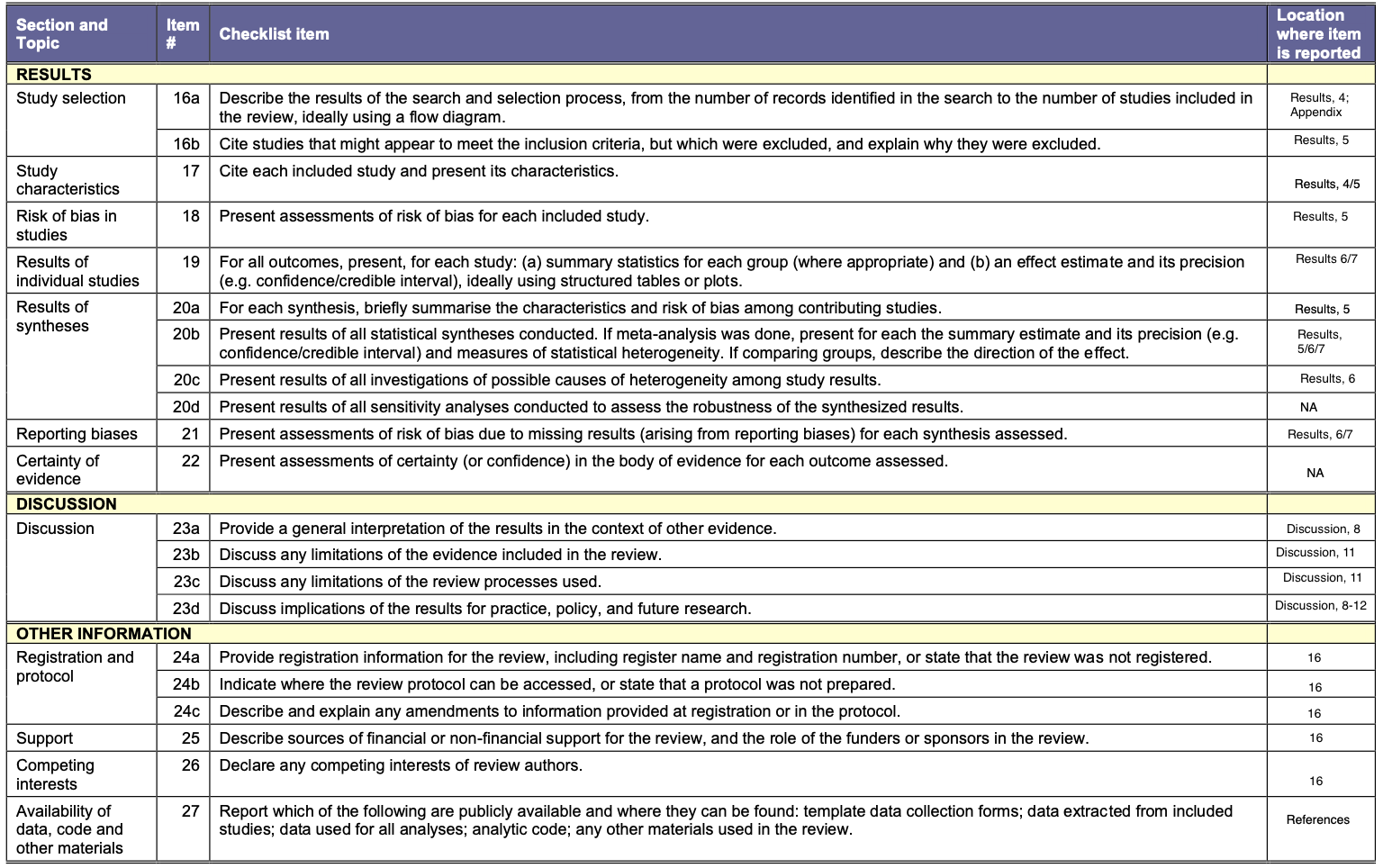


References

1. Higgins, J. P. T. *et al.* The Cochrane Collaboration’s tool for assessing risk of bias in randomised trials. *BMJ* **343**, (2011).

2. Barter, P. J. *et al.* Effect of torcetrapib on glucose, insulin, and hemoglobin a1c in subjects in the investigation of lipid level management to understand its impact in atherosclerotic events (Illuminate) Trial. *Circulation* (2011) doi:10.1161/CIRCULATIONAHA.111.018259.

3. Schwartz, G. G. *et al.* Effects of Dalcetrapib in Patients with a Recent Acute Coronary Syndrome. *N. Engl. J. Med.* (2012) doi:10.1056/nejmoa1206797.

4. Lincoff, A. M. *et al.* Evacetrapib and Cardiovascular Outcomes in High-Risk Vascular Disease. *N. Engl. J. Med.* (2017) doi:10.1056/nejmoa1609581.

5. Bowman, L. *et al.* Effects of Anacetrapib in Patients with Atherosclerotic Vascular Disease. *N. Engl. J. Med.* **377**, 1217–27 (2017).

6. Cannon, C. P. *et al.* Safety of anacetrapib in patients with or at high risk for coronary heart disease. *N. Engl. J. Med.* **363**, 2406‐2415 (2010).
